# Low Branched Chain Amino Acids and Tyrosine in Thai Patients with Type 2 Diabetes Mellitus Treated with Metformin and Metformin-Sulfonylurea Combination Therapies

**DOI:** 10.3390/jcm10225424

**Published:** 2021-11-20

**Authors:** Natthida Sriboonvorakul, Wirichada Pan-Ngum, Kittiyod Poovorawan, Sant Muangnoicharoen, Lauren M. Quinn, Bee K. Tan

**Affiliations:** 1 Department of Clinical Tropical Medicine, Faculty of Tropical Medicine, Mahidol University, Bangkok 10400, Thailand; natthida.srn@mahidol.ac.th (N.S.); kittiyod.poo@mahidol.ac.th (K.P.); sant.mua@mahidol.ac.th (S.M.); 2 Department of Tropical Hygiene, Faculty of Tropical Medicine, Mahidol University, Bangkok 10400, Thailand; wirichada.pan@mahidol.ac.th; 3 Department of Immunology and Immunotherapy, University of Birmingham, Birmingham B15 2TT, UK; l.quinn.1@bham.ac.uk; 4 Department of Cardiovascular Sciences and Diabetes Research Centre, University of Leicester, Leicester LE1 7RH, UK

**Keywords:** amino acids, liquid chromatography-mass spectrometry, LC-MS, metformin, sulfonyl-urea, type 2 diabetes mellitus, T2DM

## Abstract

Type 2 diabetes mellitus (T2DM) is a growing public health challenge for Thailand (current prevalence ~10.0%). Amino acids offer novel biomarkers to predict risk of T2DM and indicate sub-optimal disease management, which could facilitate earlier treatment. We studied amino acid profiles in a Thai cohort comprising of individuals with T2DM (*n* = 65 single-drug-treated; *n* = 38 multi-drug-treated) compared to healthy controls (*n* = 104) using liquid chromatography-mass spectrometry. Age and BMI were significantly lower in the healthy controls compared to the single or multi-treated T2DM groups. The BCAA (leucine and valine) were significantly lower in the single and multi-treated T2DM groups compared to healthy controls (*p* < 0.001 and *p* < 0.001) and isoleucine was significantly lower in the single-treated compared to the healthy controls (*p* = 0.014). These findings beg the question whether BCAAs supplementation be beneficial in T2DM patients treated with single or multi-drug therapy? Tyrosine was significantly lower in the single and multi-treated T2DM groups compared to healthy controls (*p* < 0.001 and *p* = 0.002), whereas phenylalanine was significantly higher in the multi-treated T2DM group compared to the single treated T2DM group (*p* = 0.045). We provide novel insights into the effects of diabetes treatments on these amino acids in insulin resistant states such as T2DM in a unique but understudied Thai population.

## 1. Introduction

Type 2 diabetes mellitus (T2DM) represents a major public health burden worldwide. In South East Asia, rates of T2DM have achieved epidemic proportions, with increased propensity for younger individuals and those with lower BMI to be at greater risk of T2DM compared to White European individuals. T2DM is a growing public health challenge for Thailand with a prevalence of ~10.0% (2.3% in 1991) [1]. This is may be related to increasing sugar consumption; between 1983 and 2009 sugar consumption rose from 12.7 to 31.2 kg per person per year [1]. The results of the most recent National Thai Food Consumption Survey suggest that a higher intake of protein, sugar and fat, and the increasing total calories is driving an epidemic of obesity in Thailand [2]. Insulin resistance combined with earlier beta-cell dysfunction in this population leads to a premature insulin requirement, and higher rates of diabetes related complications (cardiovascular and renal disease), which confers greater risk of mortality [3]. T2DM therefore represents a major public health burden in this region.

T2DM is characterized by hyperglycaemia and insulin resistance, but also exhibits dysregulation of protein metabolism, resulting from impaired insulin secretion and/or insulin resistance [4]. Deficiency of insulin leads to increased gluconeogenesis, glycogenolysis and protein breakdown in skeletal muscle [5], and insulin resistance is characterized by increased proteolysis and decreased protein synthesis [6]. Elevated plasma concentrations of branched chain amino acids (BCAA) including leucine, isoleucine and valine, and aromatic amino acids (AAA) including tyrosine and phenylalanine, circulate up to 10 years prior to a diagnosis of T2DM, hence interest in their role as biomarkers for insulin resistance and T2DM [6]. Cheng et al. further clarified that it was metabolically unhealthy (defined as insulin resistant, hypertensive, unfavourable adiposity, and/or pro-inflammatory) overweight or obese individuals, where BCAA and AAA were significantly higher, compared to metabolically healthy overweight or obese individuals [7].

The possibility of pharmacological treatment for T2DM, including metformin and sulfonylureas, restoring aberrant amino acid profiles has been explored [8,9,10]. The mechanism of action of metformin is still not fully understood, but it acts directly or indirectly on the liver to lower glucose production, and acts on the gut to increase glucose utilization, increase glucagon-like peptide-1 (GLP-1) and influence the gut microbiome. At the molecular level, metformin inhibits the mitochondrial respiratory chain in the liver, leading to AMP-activated protein kinase (AMPK) enhancing insulin sensitivity and lowering cyclic AMP (cAMP), thus reducing the expression of gluconeogenic enzymes. Metformin also has AMPK-independent effects on the liver that may include inhibition of fructose-1,6-bisphosphatase by AMP [11]. Alternatively, sulfonylureas act by binding to their specific receptor on β-pancreatic cells, blocking the inflow of potassium (K^+^) through the ATP-dependent channel, which depolarizes the cell membrane and prevents calcium diffusion. This mechanism triggers contraction of the actomyosin filaments, which stimulates exocytotic insulin secretion [12]. Population studies investigating the effect of metformin and sulfonylureas on amino acid profiles have demonstrated mixed results but have thus far not affirmed the anticipated restorative benefit in reducing BCAA or AAA circulating levels [8,9,10]. The aim of this study was to compare amino acid profiles for BCAA, AAA and glutamate/glutamine, in a Thai cohort with T2DM, following treatment with single (metformin) or multiple drug (metformin and sulfonylurea) therapy, compared to healthy controls.

## 2. Materials and Methods

We performed liquid chromatography-mass spectrometry (LC-MS) analysis for quantification of amino acids in plasma samples, comparing amino acid profiles across three groups: healthy controls, compared to single-treated vs multi-treated individuals with T2DM.

### 2.1. Study Participants

The study was conducted according to the guidelines of the Declaration of Helsinki and approved by the Bangkok Hospital for Tropical Diseases in Thailand certified by the Ethics Committee of the Faculty of Tropical Medicine, Mahidol University (protocol number MUTM 2015-069, date of approval 27 November 2018). Written informed consent was obtained from all study participants. Please see Supplementary Figure S1 for the study flow chart.

All Thai adult participants were recruited in the Hospital for Tropical Diseases, Faculty of Tropical Medicine, Thailand. Inclusion criteria was age ≥35 to ≤65 years. Pregnant women were excluded. The modified World Health Organization (WHO) criteria (2018) was used to define individuals with T2DM [13]. All T2DM study participants had been diagnosed using a single fasting plasma glucose (FPG) ≥ 126 mg/dL with a history of the classic symptoms of T2DM such as increased thirst (polydipsia) and frequent urination (polyuria) or a repeat FPG of ≥126 mg/dL in those with no symptoms.. Healthy controls were classified by an FPG <100 mg/dL and with no history of elevated FPG. T2DM patients were divided into two groups according to diabetes medication; single treated with Metformin alone, and multi-treated (metformin and sulfonylurea) and the participant cohort had no history of significant diabetes complications. Please see Supplementary Data 1 for calculation of the sample size of the study.

### 2.2. Sample Collection and Preparation

Baseline clinical and laboratory data of the participants were obtained in the hospital (i.e., glucose, HbA1c and lipid profile). Fasting venous blood was collected from all participants and was then divided into four aliquots; the first aliquot was whole blood collected in an EDTA tube for HbA1c analysis, the second aliquot was collected in a sodium fluoride tube and centrifuged for 7 min at 2500× *g* at room temperature to prepare plasma for glucose analysis and the third aliquot was collected in a clot blood tube and centrifuged for 7 min at 2500× *g* at room temperature to prepare serum for creatinine and lipid profile (cholesterol, triglyceride and high-density lipoprotein (HDL). All blood chemistry analyses were performed using a Cobas^®^ c501 Chemistry analyser, (Roche, Basel, Switzerland) in the hospital laboratory. Low-density lipoprotein (LDL) levels were calculated using the Friedewald equation. The fourth aliquot was collected in an EDTA tube and centrifuged for 7 min at 2500× *g* at room temperature to prepare plasma that was stored at −80 °C until analysis of the amino acids by liquid chromatography-mass spectrometry (LC-MS).

### 2.3. Liquid Chromatography-Mass Spectrometry (LC-MS)

The analytical method for quantifying amino acids in human plasma has been described previously [8]. Amino acids were extracted from individual plasma samples for analysis using a PhenomenexEZ: faast™ amino acid analysis kit (Phenomenex, Torrance, CA, USA) as described [14]. Plasma free amino acids were separated and quantified by LC-MS using an API 5000 Triple Quadrupole Mass Spectrometer (SCIEX, Framingham, MA, USA). Reversed-phase chromatographic separation was performed on an EZ: fasst^TM^ AAA-MS™ column (250 × 3.0 mm, Phenomenex) at 35 °C and a flow rate of 250 µL/min with a total run time of 17 min. The mobile phase consisted of an A-B mixture of 10 mM ammonium formate in water (A) and 10 mM ammonium formate in methanol (B), where the methanol percentage was changed linearly as follows: 0 min, 68%; 2.5 min, 83%; 10 min, 83%; 17 min, 68%. A sample volume of 1 µL was injected and detection was performed using tandem MS with multiple reaction monitoring (MRM) transitions in the positive ion mode with electro-spray ionization. The MRM method was set fixed Q1 and Q3 for mass scanning and consisted of three periods of analysis, which had a total of 20 MRMs. Prior to data acquisition, we optimized the LC system to detect the internal standards (homoarginine, methionine-d3, homophenylalanine). The scan range was 50–600 *m*/*z*. Inter-assay variation scores were determined for the three amino acid standards. Quantitative data was obtained from calibration standards based on a linear standard curve using MultiQuant (AB SCIEX™, Framingham, MA, USA). Quantification of the amino acids was monitored and controlled for outliers using the Food and Drug Administration (FDA) guideline. The amino acids measurements were within the valid range. Measurements below the detectable level were excluded from the analyses. There were 3 missing values that were excluded in our data analyses. We used an internal standard to adjust for any loss of analyte during sample preparation and compensate for signals with high coefficient of variation (CV). We performed experiments in three batches. We used quality-controlled samples, which covered the expected study sample concentration ranges (low, middle and high concentrations); the samples were prepared in standardised conditions according to our study aims and protocols.

### 2.4. Statistical Analysis

Data was found to be not normally distributed on the Kolmogornov-Smirnov, Shapiro-Wilk, and D’Agostino and Pearson normality tests (apart from leucine, tyrosine, arginine, and methionine which were normally distributed). Parametric and non-parametric statistical tests were used accordingly. Descriptive data are presented as median +/− interquartile range. Within groups, Spearman’s rank correlation was performed to compare inter-relationships between amino acids and demographic factors. Following this, regression analyses were performed on the significantly correlated amino acids, which were square root transformed variables to perform linear regression, to identify independent predictors. Further, comparisons were drawn between the single treated, and multi-treated T2DM and healthy controls. Kruskal Wallis and ANOVA were used to compared differences between the three groups, with Bonferroni correction post-hoc analysis used for between group comparisons. Non-parametric variables were transformed to the square root in light of extreme negative skew. ANCOVA was then applied to adjust for age and BMI as covariates. Statistical analyses were conducted using GraphPad PRISM® version 9.0 (GraphPad Software Inc., San Diego, CA, USA) and SPSS version 28.0 (IBM, Armonk, NY, USA). The level of significance was set at *p* < 0.05.

## 3. Results

Table 1 shows the characteristics of the three groups i.e., healthy controls (G0), T2DM treated with metformin (G1) and T2DM treated with metformin and sulfonylurea (G2). Median age was 51 years (interquartile range: 43–57 years) and was significantly lower in the healthy controls [48 (41–54) years] compared to the diabetes treated groups [55 (49–58) years] and [53 (47–57) years]. The cohort was a South East Asian (Thai) population and 58 (28%) were male. BMI was also significantly lower in the healthy controls [G0: 24.5 (21.8–27.1) kg/m^2^] compared to the diabetes treated groups, [G1: 27.8 (25.7–31.6) kg/m^2^; G2: 27.0 (24.8–30.4) kg/m^2^]. We found no significant differences in demographics in the single (G1) vs the multi-treated (G2) T2DM groups and HbA1c was comparable but above target in both T2DM groups.

### 3.1. Amino Acid Correlations and Regression Analysis

Table 2 and Table 3 show the correlation between BCAA, AAA, glutamate and glutamine, using Spearman’s rank test, for the healthy controls, single-treated, and multi-treated T2DM groups. Importantly, the BCAA were generally highly correlated with each other (isoleucine, leucine and valine), across all groups. See Supplementary Table S1 for the full list of amino acid correlations. Regression analysis revealed unique predictors for each amino acid (Table 4). The strong correlation between the amino acids suggests a common but controlled relationship, in terms of their biological interactions in vivo, related in part to their structural characteristics, with implications for the further understanding of the interplay and regulation of these amino acids in both health and disease, in particular, insulin resistant states and T2DM.

### 3.2. Amino Acid Profile between Healthy Controls, Single and Multi-Treated T2DM Groups

Table 5 shows the amino acid profiles of the healthy controls (G0), the single (G1) and multi-treated (G2) T2DM groups.

#### 3.2.1. Branched Chain Amino Acids

In both of the T2DM treated groups, i.e., G1 and G2, BCAA were significantly lower than the healthy controls (G0). Isoleucine was significantly lower in the single-treated T2DM group compared to the healthy controls (adjusted *p* = 0.014 with Bonferroni correction). Valine was significantly lower in both the T2DM treated groups (single or multiple) compared to healthy controls (adjusted *p* < 0.0001 and *p* = 0.001 with Bonferroni correction, respectively). Leucine was significantly lower in both T2DM treated groups (single or multiple-treated) compared to healthy controls (*p* < 0.0001 and *p* < 0.0001 with Bonferroni correction, respectively). After adjustment for age and BMI, leucine remained significantly different between the T2DM treated groups and the healthy controls.

#### 3.2.2. Aromatic Amino Acids

Phenylalanine was significantly higher in the multi-treated T2DM group compared to the single treated T2DM group (adjusted *p* = 0.045 with Bonferroni correction). Tyrosine was significantly lower in both the single and multi-treated T2DM groups compared to healthy controls (adjusted *p* < 0.0001 and *p* = 0.002 with Bonferroni correction, respectively).

#### 3.2.3. Glutamate and Glutamine

Glutamate was significantly lower in the multi-treated T2DM group compared to the single treated T2DM group (adjusted *p* = 0.002). Glutamine was significantly lower in both the single treated and multi-treated T2DM groups compared to the healthy controls (adjusted *p* < 0.0001 and *p* < 0.0001 with Bonferroni correction, respectively). The glutamate/glutamine ratio was highest in the single treated T2DM group and lower in the healthy controls and multi-treated T2DM.

## 4. Discussion

We show that BCAA (leucine and valine), tyrosine and glutamine were all significantly lower in the single or multi-treated individuals with T2DM compared to the healthy controls. Isoleucine was significantly lower in the single-treated T2DM group compared to healthy controls, and this remained significant after adjustment for age and BMI. We also show that phenylalanine was higher in the multi-treated individuals with T2DM compared to single treated and healthy controls. Glutamate was significantly lower in multi-treated compared to single-treated T2DM. Glutamine and glutamate were significant predictors of each other in the T2DM groups.

### 4.1. Branched Chain Amino Acids

BCAA as biomarkers in Thai individuals has not been previously demonstrated. Tillin et al. compared amino acid profiles in White European compared to South Asians over 19 years follow-up and found that South Asians had higher serum isoleucine, tyrosine and phenylalanine concentrations, and greater risk of developing T2DM but there was a weaker association with obesity, compared to White European [15]. Tai et al. reported that Chinese individuals with a higher homeostasis model assessment (HOMA) had significantly higher BCAA than individuals with a lower HOMA index [16]. Lee et al. showed at five years follow-up that BCAA was significantly and positively correlated with T2DM in White European and Hispanic individuals but not for African Americans [17]. Whether BCAA principally causes an insulin resistant state or the reductions in branched-chain α-ketoacid dehydrogenase (BCKD) and tyrosine aminotransferase (TAT) observed in insulin resistant states is responsible for the elevated BCAA is not well delineated. BCAA infusion is shown to reduce insulin sensitivity [18], whereas BCAA deprivation improves insulin sensitivity in vivo [19]. In our healthy control cohort with normoglycaemia, the higher levels of BCAA observed warrant investigation in prospective studies to ascertain their predictive abilities as biomarkers of T2DM and complications in a Thai cohort, in light of their significant risk associations in high-risk cohorts such as South Asians.

### 4.2. Aromatic Amino Acids

In a large Asian (Japanese) cohort, AAAs were associated with lipids and obesity [20], and Tillin et al. showed in South Asian non-diabetic men that AAAs were higher compared to White European [15]. Both the CAMERA [10] and CIMT [21] trials showed reductions in tyrosine following metformin therapy which supports our study’s findings. Most importantly, the ADVANCE study was performed in patients with T2DM and found phenylalanine was positively associated with risk of macrovascular disease, following adjustment for age, sex and randomized group (perindopril/indapamide vs gliclazide vs standard care [22]. Here, tyrosine was also inversely associated with microvascular disease risk [22]. Therefore, the higher phenylalanine in the multi-treated T2DM group and the lower tyrosine in both T2DM groups observed in our study, support the role of AAAs as biomarkers for macro and microvascular disease respectively, and warrant further investigation in prospective studies in South East Asian populations.

### 4.3. Glutamate and Glutamine

Finally, glutamate and glutamine are also associated with T2DM risk [23,24]; glutamate is higher in patients with T2DM and pre-diabetes whereas glutamine has been negatively associated [23]. A lower glutamine to glutamate ratio (G/G) has also been associated with the incidence of T2DM [25]. Rhee et al. showed that glutamine and glutamate were the best predictors of diabetic retinopathy [26]. The Diabetes Prevention study showed no change in the G/G ratio following 2 years of metformin therapy [27], but in our study G/G ratio was highest in the single treated group; prospective studies are therefore required to determine the direction of change and crucially to identify causal mechanisms.

### 4.4. Metformin Therapy

In this study, we report reductions in BCAAs in single and multi-treated individuals with T2DM. Metformin has been shown to restore the BCAA profile in vivo; Zemdegs et al. showed that metformin normalized BCAA levels in insulin resistant mice [28], and Riera-Borrull et al. showed that BCAA decreased following metformin therapy in mice fed a high-fat diet [29]. Irving et al. suggested metformin upregulated BCAA catabolic enzyme activity, as a restorative mechanism to lower BCAA levels in insulin resistant states [30].

Conversely, population studies have demonstrated mixed findings. The CIMT trial performed in a Danish T2DM cohort compared amino acid profiles between metformin and placebo treatment [21]; eighty-four percent (*n* = 312/370) of participants were treated with metformin prior to the study [21]. From these individuals, leucine and isoleucine were higher and valine was lower in individuals previously receiving metformin treatment [21]. After 18 months follow-up, valine further decreased and isoleucine/leucine further increased in the metformin treated groups, and although there was a reduction in HbA1c in both groups, this remained out of target range [21]. None of the amino acids were predictors of the glucose lowering effect of metformin [21]. However, the Diabetes Prevention Programme was a multi-ethnic trial (54.7% White European) comparing metformin or lifestyle intervention to placebo in people with pre-diabetes or diabetes, and showed no difference in BCAA, AAA or G/G ratio between intervention groups at 2 years follow-up [27]. Further, the CAMERA study randomized individuals with coronary artery disease but no T2DM to metformin or placebo and found no change in BCAA following metformin therapy [10]. Bariatric surgery significantly decreases both BCAA and AAA [31], so the amino acid profiles observed may reflect the degree of insulin resistance vs insulin sensitivity, which has not been well delineated in the metformin studies.

In our Thai cohort receiving metformin monotherapy, the reduction in valine, may represent a marker of treatment benefit with metformin, as shown in the CIMT trial [21]. The lower levels of isoleucine and leucine may be explained by differences in the characteristics of study populations, in particular, genetic and environmental differences. Nevertheless, further investigations are pertinent as the ADVANCE trial reported inverse associations between valine and leucine with mortality [22], and Saleem et al. showed that BCAA were lower in individuals with micro or macroalbuminuria [6].

### 4.5. Metformin and Sulfonylurea Therapy

Walford et al. compared amino acid profiles in insulin resistant (IR) vs insulin sensitive (IS) individuals, treated with metformin or glipizide [32]. Overall, BCAA and AAA were lower in the IS than the IR participants [32]. Following glipizide treatment, all BCAA and AAA significantly decreased in the IS group, but there were no significant changes in the IR participants [32]. There were no significant changes in BCAA or AAA in the IS group following metformin treatment, but there was a significant increase in leucine and isoleucine in the IR group [32]. Irving et al. compared combined pioglitazone and metformin treatment to placebo in a White European T2DM cohort and found significant reductions in phenylalanine and tyrosine, 3 months following treatment [30], but there was no dose response effect between insulin sensitivity and reductions in amino acids, and no change in the BCAA [33]. Pioglitazone was previously reported to decrease BCAA (valine and lysine) in T2DM patients through increased activation of BCKD in adipose tissues [33], and Bao et al. showed rosiglitazone significantly lowered BCAA following 6-months of therapy in T2DM patients [34]. The increase in phenylalanine with multi-treatment and the reductions in BCAA may reflect differing pharmacokinetics in East Asian individuals.

### 4.6. Significance of Low Branched Chain Amino Acids and Tyrosine

The BCAAs (valine, leucine and isoleucine) are essential amino acids; BCAAs stimulate anabolic pathways and thus mitigate cachexia, prevent hepatic encephalopathy, lower fatigue during exercise, stimulate wound healing and promote insulin production [35]. As mentioned above, BCAAs are positively correlated with insulin resistance and T2DM [15,16,17]; this could be a compensatory homeostatic reaction to deal with insulin resistance by stimulating insulin production. The results presented in this study show that normoglycaemic controls have higher BCAA levels than T2DM patients receiving either metformin or a combination of metformin and sulfonylurea (plausibly a secondary effect in our Thai population). Therefore, the low levels of BCAAs observed in our study begs the question whether BCAAs supplementation may be beneficial in Thai patients with T2DM treated with either metformin or a combination of metformin and sulfonylurea.

On the other hand, tyrosine is metabolized to 3,4-dihydroxy-ʟ-phenylalanine (ʟ-DOPA) and dopamine (DA) in the foregut; ʟ-DOPA and DA are inhibitors of beta cell glucose-stimulated insulin secretion. Hence, low tyrosine levels observed in our study could be beneficial to Thai patients with T2DM treated with either metformin or a combination of metformin and sulfonylurea [36].

Dietary data, a potential confounder, was not collected; however, a recent systematic review have shown no positive effects of dietary intake on amino acids [37]. We recommend further studies to strengthen our observations. Whether glucose homeostasis is achieved with pharmacological or lifestyle intervention is also an important consideration when determining the direction of change in amino acid profiles in future studies.

## 5. Conclusions

We have demonstrated that BCAA and tyrosine are reduced in Thai patients with T2DM treated with either metformin or metformin-sulfonylurea combination therapies. We also show strong relationships between the 20 amino acids for the healthy controls, single-treated, and multi-treated T2DM groups, which would aid future research into the interplay and regulation of these amino acids in both health and disease, in particular, T2DM.

## Figures and Tables

**Table 1:** Characteristics of study subjects.

Variable	G0 (*n* = 104)	G1 (*n* = 65)	G2 (*n* = 38)	*p* Value
Gender (women/men)	85/19	38/27	26/12	
Age (year)	48.0 (51.0–54.0)	55.0 (49.0–58.0)	53.0 (47.0–57.0)	<0.001
BMI (kg/m^2^)	24.5 (21.8–27.1)	27.8 (25.7–31.6)	27.0 (24.8–30.4)	<0.001
Glucose (mg/dL)	89.0 (84.0–95.0)	132.0 (113.0–161.0)	129.0 (112.0–179.0)	<0.001
HbA1c (%)	5.4 (5.1–5.5)	6.9 (6.2–7.6)	6.8 (6.0–8.3)	<0.001
Total-C (mg/dL)	205.0 (180.0–227.0)	176.0 (163.0–210.0)	185.0 (158.0–212.0)	<0.001
HDL-C (mg/dL)	62.0 (54.0–71.0)	50.0 (44.0–59.0)	53.0 (47.0–66.0)	<0.001
LDL-C (mg/dL)	120.0 (96.0–142.0)	94.0 (78.0–114.0)	100.0 (71.0–115.0)	<0.001
Triglyceride (mg/dL)	97.0 (73.0–128.0)	135.0 (104.0–168.0)	129.0 (94.0–172.0)	<0.001
Creatinine (mg/dL)	0.72 (0.63–0.84)	0.78 (0.64–0.92)	0.73 (0.59–0.95)	<0.001
eGFR (ml/min/1.73m²)	99.5 (88.8–107.0)	95.0 (83.8–103.0)	100.5 (89.3–105.8)	0.329

Data are median (interquartile range). BMI = body mass index; HbA1C = glycated haemoglobin; Total-C = total cholesterol; HDL = High-density lipoprotein; LDL = Low-density lipoprotein; eGFR = estimated Glomerular Filtration Rate.

**Table 2:** Correlation coefficient (r) and significance (*p*) using the spearman’s rank correlation test for healthy controls (G0), single-treated (G1) and multi-treated (G2) type 2 diabetes mellitus groups for the Branched chain amino acids (BCAA). * Correlation is significant at the 0.05 level (2-tailed); ** Correlation is significant at the 0.01 level (2-tailed).

Amino Acid (nM)	Spearman’s Rank Correlation	G0			G1			G2		
		Isoleucine (nM)	Valine (nM)	Leucine (nM)	Isoleucine (nM)	Valine (nM)	Leucine (nM)	Isoleucine (nM)	Valine (nM)	Leucine (nM)
Isoleucine	Correlation Coefficient	1.000	0.795 **	0.828 **	1.000	0.728 **	0.757 **	1.000	0.795 **	0.828 **
	Significance (*p* value)		**0.000**	**0.000**		**0.000**	**0.000**		**0.000**	**0.000**
Valine	Correlation Coefficient	0.795 **	1.000	0.902 **	0.728 **	1.000	0.839 **	0.795 **	1.000	0.902 **
	Significance (*p* value)	**0.000**		**0.000**	**0.000**		**0.000**	**0.000**		**0.000**
Leucine	Correlation Coefficient	0.828 **	0.902 **	1.000	0.757 **	0.839 **	1.000	0.828 **	0.902 **	1.000
	Significance (*p* value)	**0.000**	**0.000**		**0.000**	**0.000**		**0.000**	**0.000**	
Phenylalanine	Correlation Coefficient	0.618 **	0.203	0.247	0.400 **	−0.098	0.063	0.618 **	0.203	0.247
	Significance (*p* value)	**0.000**	0.221	0.135	**0.001**	0.439	0.619	**0.000**	0.221	0.135
Tyrosine	Correlation Coefficient	−0.163	0.072	0.096	0.050	0.350 **	0.438 **	−0.163	0.072	0.096
	Significance (*p* value)	0.327	0.667	0.566	0.694	**0.004**	**0.000**	0.327	0.667	0.566
Aminoadipate	Correlation Coefficient	0.420 **	0.361 *	0.316	0.301 *	0.197	0.112	0.420 **	0.361 *	0.316
	Significance (*p* value)	**0.009**	**0.026**	0.053	**0.015**	0.115	0.376	**0.009**	**0.026**	0.053
Arginine	Correlation Coefficient	0.148	0.219	0.406 *	0.083	0.182	0.300 *	0.148	0.219	0.406 *
	Significance (*p* value)	0.374	0.187	**0.011**	0.509	0.148	**0.015**	0.374	0.187	**0.011**
Glycine	Correlation Coefficient	0.659 **	0.570 **	0.480 **	0.411 **	0.380 **	0.236	0.659 **	0.570 **	0.480 **
	Significance (*p* value)	**0.000**	**0.000**	**0.002**	**0.001**	**0.002**	0.061	**0.000**	**0.000**	**0.002**
Threonine	Correlation Coefficient	0.474 **	0.326 *	0.223	0.265 *	0.089	−0.089	0.474 **	0.326 *	0.223
	Significance (*p* value)	**0.003**	**0.045**	0.179	**0.033**	0.482	0.481	**0.003**	**0.045**	0.179
Methionine	Correlation Coefficient	0.386 *	0.434 **	0.479 **	0.253 *	0.282 *	0.442 **	0.386 *	0.434 **	0.479 **
	Significance (*p* value)	**0.017**	**0.007**	**0.002**	**0.042**	**0.023**	**0.000**	**0.017**	**0.007**	**0.002**
Aspartate	Correlation Coefficient	0.115	0.068	0.158	0.347 **	0.307 *	0.359 **	0.115	0.068	0.158
	Significance (*p* value)	0.492	0.686	0.343	**0.005**	**0.014**	**0.004**	0.492	0.686	0.343
Sacosine	Correlation Coefficient	−0.517 **	−0.640 **	−0.521 **	−0.403 **	−0.337 **	−0.126	−0.517 **	−0.640 **	−0.521 **
	Significance (*p* value)	**0.001**	**0.000**	**0.001**	**0.001**	**0.006**	0.317	**0.001**	**0.000**	**0.001**
Ornithine	Correlation Coefficient	−0.152	0.073	0.026	−0.053	0.110	0.204	−0.152	0.073	0.026
	Significance (*p* value)	0.363	0.662	0.875	0.674	0.381	0.104	0.363	0.662	0.875
Proline	Correlation Coefficient	0.620 **	0.875 **	0.777 **	0.670 **	0.667 **	0.555 **	0.620 **	0.875 **	0.777 **
	Significance (*p* value)	**0.000**	**0.000**	**0.000**	**0.000**	**0.000**	**0.000**	**0.000**	**0.000**	**0.000**
Lysine	Correlation Coefficient	−0.344 *	−0.034	0.047	−0.141	0.104	0.327 **	−0.344 *	−0.034	0.047
	Significance (*p* value)	**0.034**	0.838	0.778	0.263	0.409	**0.008**	**0.034**	0.838	0.778
Glutamate	Correlation Coefficient	−0.241	−0.098	0.001	−0.022	0.141	0.317 **	−0.241	−0.098	0.001
	Significance (*p* value)	0.146	0.557	0.995	0.860	0.262	**0.010**	0.146	0.557	0.995
Glutamine	Correlation Coefficient	0.062	0.169	0.163	−0.059	0.176	0.266 *	0.062	0.169	0.163
	Significance (*p* value)	0.711	0.309	0.328	0.642	0.160	**0.033**	0.711	0.309	0.328
Serine	Correlation Coefficient	0.422 **	0.280	0.143	0.195	0.005	−0.141	0.422 **	0.280	0.143
	Significance (*p* value)	**0.008**	0.088	0.391	0.119	0.968	0.261	**0.008**	0.088	0.391
Asparagine	Correlation Coefficient	0.474 **	0.379 *	0.258	0.228	0.156	−0.007	0.474 **	0.379 *	0.258
	Significance (*p* value)	**0.003**	**0.019**	0.117	0.068	0.216	0.953	**0.003**	**0.019**	0.117
4-Hydroxy-proline	Correlation Coefficient	0.321 *	0.122	0.048	0.176	−0.006	−0.211	0.321 *	0.122	0.048
	Significance (*p* value)	**0.049**	0.465	0.773	0.161	0.961	0.091	**0.049**	0.465	0.773

**Table 3:** Correlation coefficient (r) and significance (*p*) using the spearman’s rank correlation test for healthy controls (G0), single-treated (G1) and multi-treated (G2) type 2 diabetes mellitus groups for the Aromatic amino acids (AAA), glutamate and glutamine. * Correlation is significant at the 0.05 level (2-tailed); ** Correlation is significant at the 0.01 level (2-tailed).

Amino Acid (nM)	Spearman’s Rank Correlation	G0				G1				G2			
		Phenylalanine (nM)	Tyrosine (nM)	Glutamate (nM)	Glutamine (nM)	Phenylalanine (nM)	Tyrosine (nM)	Glutamate (nM)	Glutamine (nM)	Phenylalanine (nM)	Tyrosine (nM)	Glutamate (nM)	Glutamine (nM)
Isoleucine	Correlation Coefficient	0.618 **	−0.163	−0.241	0.062	0.400 **	0.050	−0.022	−0.059	0.618 **	−0.163	−0.241	0.062
	Significance (*p* value)	**0.000**	0.327	0.146	0.711	**0.001**	0.694	0.860	0.642	**0.000**	0.327	0.146	0.711
Valine	Correlation Coefficient	0.203	0.072	−0.098	0.169	−0.098	0.350 **	0.141	0.176	0.203	0.072	−0.098	0.169
	Significance (*p* value)	0.221	0.667	0.557	0.309	0.439	**0.004**	0.262	0.160	0.221	0.667	0.557	0.309
Leucine	Correlation Coefficient	0.247	0.096	0.001	0.163	0.063	0.438 **	0.317 **	0.266 *	0.247	0.096	0.001	0.163
	Significance (*p* value)	0.135	0.566	0.995	0.328	0.619	**0.000**	**0.010**	**0.033**	0.135	0.566	0.995	0.328
Phenyl-alanine	Correlation Coefficient	1.000	−0.425 **	−0.506 **	−0.158	1.000	−0.370 **	−0.310 *	−0.371 **	1.000	−0.425 **	−0.506 **	−0.158
	Significance (*p* value)		**0.008**	**0.001**	0.343		**0.002**	**0.012**	**0.002**		**0.008**	**0.001**	0.343
Tyrosine (nm)	Correlation Coefficient	−0.425 **	1.000	0.396 *	0.386 *	−0.370 **	1.000	0.440 **	0.629 **	−0.425 **	1.000	0.396 *	0.386 *
	Significance (*p* value)	**0.008**		**0.014**	**0.017**	**0.002**		**0.000**	**0.000**	**0.008**		**0.014**	**0.017**
Amino-adipate	Correlation Coefficient	0.352 *	0.115	−0.274	0.082	0.302 *	0.013	−0.256 *	−0.181	0.352 *	0.115	−0.274	0.082
	Significance (*p* value)	**0.030**	0.492	0.095	0.624	**0.015**	0.918	**0.040**	0.148	**0.030**	0.492	0.095	0.624
Arginine	Correlation Coefficient	−0.285	0.385 *	0.361 *	0.080	−0.244	0.441 **	0.489 **	0.271 *	−0.285	0.385 *	0.361 *	0.080
	Significance (*p* value)	0.083	**0.017**	**0.026**	0.633	0.051	**0.000**	**0.000**	**0.029**	0.083	**0.017**	**0.026**	0.633
Glycine	Correlation Coefficient	0.510 **	−0.073	−0.291	0.404 *	0.216	−0.001	−0.203	0.205	0.510 **	−0.073	−0.291	0.404 *
	Significance (*p* value)	**0.001**	0.665	0.076	**0.012**	0.087	0.994	0.107	0.103	**0.001**	0.665	0.076	**0.012**
Threonine	Correlation Coefficient	0.488 **	−0.104	−0.347 *	0.504 **	0.282 *	−0.179	−0.336 **	0.210	0.488 **	−0.104	−0.347 *	0.504 **
	Significance (*p* value)	**0.002**	0.536	**0.033**	**0.001**	**0.023**	0.153	**0.006**	0.093	**0.002**	0.536	**0.033**	**0.001**
Methionine	Correlation Coefficient	0.091	0.441 **	0.180	0.448 **	0.027	0.481 **	0.303 *	0.336 **	0.091	0.441 **	0.180	0.448 **
	Significance (*p* value)	0.588	**0.006**	0.278	**0.005**	0.829	**0.000**	**0.014**	**0.006**	0.588	**0.006**	0.278	**0.005**
Aspartate	Correlation Coefficient	0.033	0.145	0.420 **	−0.049	0.137	0.090	0.337 **	−0.009	0.033	0.145	0.420 **	−0.049
	Significance (*p* value)	0.843	0.384	**0.009**	0.772	0.280	0.479	**0.006**	0.942	0.843	0.384	**0.009**	0.772
Sacosine	Correlation Coefficient	−0.129	0.109	0.127	−0.028	−0.153	0.344 **	0.282 *	0.373 **	−0.129	0.109	0.127	−0.028
	Significance (*p* value)	0.439	0.516	0.449	0.867	0.223	**0.005**	**0.023**	**0.002**	0.439	0.516	0.449	0.867
Ornithine	Correlation Coefficient	−0.217	0.492 **	0.421 **	0.480 **	−0.184	0.644 **	0.217	0.485 **	−0.217	0.492 **	0.421 **	0.480 **
	Significance (*p* value)	0.190	**0.002**	**0.008**	**0.002**	0.143	**0.000**	0.083	**0.000**	0.190	**0.002**	**0.008**	**0.002**
Proline	Correlation Coefficient	−0.004	0.216	0.074	0.295	0.038	0.065	0.067	−0.078	−0.004	0.216	0.074	0.295
	Significance (*p* value)	0.983	0.193	0.657	0.072	0.761	0.607	0.596	0.536	0.983	0.193	0.657	0.072
Lysine	Correlation Coefficient	−0.641 **	0.442 **	0.735 **	0.370 *	−0.419 **	0.613 **	0.538 **	0.506 **	−0.641 **	0.442 **	0.735 **	0.370 *
	Significance (*p* value)	**0.000**	**0.005**	**0.000**	**0.022**	**0.001**	**0.000**	**0.000**	**0.000**	**0.000**	**0.005**	**0.000**	**0.022**
Glutamate	Correlation Coefficient	−0.506 **	0.396 *	1.000	0.394 *	−0.310 *	0.440 **	1.000	0.385 **	−0.506 **	0.396 *	1.000	0.394 *
	Significance (*p* value)	**0.001**	**0.014**		**0.014**	**0.012**	**0.000**		**0.002**	**0.001**	**0.014**		**0.014**
Glutamine	Correlation Coefficient	−0.158	0.386 *	0.394 *	1.000	−0.371 **	0.629 **	0.385 **	1.000	−0.158	0.386 *	0.394 *	1.000
	Significance (*p* value)	0.343	**0.017**	**0.014**		**0.002**	**0.000**	**0.002**		0.343	**0.017**	**0.014**	
Serine	Correlation Coefficient	0.515 **	−0.215	−0.351 *	0.426 **	0.307 *	−0.226	−0.390 **	0.149	0.515 **	−0.215	−0.351 *	0.426 **
	Significance (*p* value)	**0.001**	0.195	**0.031**	**0.008**	**0.013**	0.070	**0.001**	0.237	**0.001**	0.195	**0.031**	**0.008**
Asparagine	Correlation Coefficient	0.458 **	−0.158	−0.367 *	0.442 **	0.109	−0.006	−0.262 *	0.325 **	0.458 **	−0.158	−0.367 *	0.442 **
	Significance (*p* value)	**0.004**	0.344	**0.023**	**0.005**	0.390	0.962	**0.035**	**0.008**	**0.004**	0.344	**0.023**	**0.005**
4−Hydroxyproline	Correlation Coefficient	0.462 **	−0.219	−0.306	0.454 **	0.347 **	−0.287 *	−0.446 **	−0.010	0.462 **	−0.219	−0.306	0.454 **
	Significance (*p* value)	**0.004**	0.187	0.061	**0.004**	**0.005**	**0.020**	**0.000**	0.939	**0.004**	0.187	0.061	**0.004**

**Table 4:** Regression analysis showing the AAs that are independent predictors (including age and BMI as independent variables); B coefficient (β) and significance (*p*); healthy controls (G0), single-treated (G1) and multi-treated (G2) type 2 diabetes mellitus groups.

**G0**			**G1**			**G2**		
**Isoleucine**								
**Independent Predictors**	***β***	***p***	**Independent Predictors**	***β***	***p***	**Independent Predictors**	***β***	***p***
Leucine	0.880	0.000	Leucine	0.625	0.000	leucine	0.747	0.002
Phenylalanine	0.253	0.001	Phenylalanine	0.329	0.000	phenylalanine	0.298	0.044
Threonine	0.145	0.010	Arginine	−0.252	0.034	lysine	−0.838	0.046
Serine	−0.236	0.000	Threonine	0.190	0.049			
			Proline	0.215	0.021			
**G0**			**G1**			**G2**		
**valine**								
**Independent predictors**	***β***	***p***	**Independent predictors**	***β***	***p***	**Independent predictors**	***β***	***p***
Leucine	0.661	0.000	Leucine	1.033	0.000	Threonine	−0.616	0.021
Phenylalanine	−0.400	0.000				Ornithine	−1.227	0.029
Sarcosine	0.240	0.000				Proline	1.168	0.001
BMI	0.799	0.008				Asparagine	0.810	0.017
						4−Hydroxyproline	−0.993	0.010
**G0**			**G1**			**G2**		
**leucine**								
**Independent predictors**	***β***	***p***	**Independent predictors**	***β***	***p***	**Independent predictors**	***β***	***p***
Phenylalanine	0.145	0.024	Arginine	0.294	0.011	(constant)	−8.991	0.039
Serine	0.130	0.018	Threonine	−0.206	0.028	Lysine	0.913	0.009
Isoleucine	0.554	0.000	Lysine	0.427	0.013	Isoleucine	0.554	0.002
Valine	0.292	0.000	Isoleucine	0.605	0.000			
			Valine	0.263	0.000			
**G0**			**G1**			**G2**		
**phenylalanine**								
**Independent predictors**	***β***	***p***	**Independent predictors**	***β***	***p***	**Independent predictors**	***β***	***p***
(Constant)	6.469	0.030	Proline	−0.376	0.014	Proline	−1.403	0.001
Methionine	0.928	0.002	Asparagine	−0.813	0.004	Isoleucine	0.693	0.044
Aspartate	−1.147	0.044	Isoleucine	0.905	0.000			
Asparagine	−0.471	0.010						
Isoleucine	0.459	0.001						
Valine	−0.508	0.000						
Leucine	0.417	0.024						
**G0**			**G1**			**G2**		
**tyrosine**								
**Independent predictors**	***β***	***p***	**Independent predictors**	***β***	***p***	**Independent predictors**	***β***	***p***
Aminoadipate	0.699	0.006	Ornithine	0.764	0.000			
Methionine	0.665	0.000	Glutamate	0.191	0.013			
Aspartate	−0.687	0.050						
Proline	−0.117	0.043						
Glutamate	0.142	0.003						
Glutamine	0.267	0.000						
Age	0.317	0.024						
BMI	0.425	0.045						
**G0**			**G1**			**G2**		
**glutamate**								
**Independent predictors**	***β***	***p***	**Independent predictors**	***β***	***p***	**Independent predictors**	***β***	***p***
Arginine	0.386	0.009				(constant)	−29.084	0.041
Methionine	−0.856	0.045				Threonine	−1.310	0.026
Aspartate	3.079	0.000				Lysine	2.510	0.032
Glutamine	−0.334	0.001				Glutamine	1.110	0.010
Tyrosine	0.709	0.003						
**G0**			**G1**			**G2**		
**glutamine**								
**Independent predictors**	***β***	***p***	**Independent predictors**	***β***	***p***	**Independent predictors**	***β***	***p***
Aminoadipate	−1.580	0.008	Sarcosine	0.177	0.033	Glutamate	0.285	0.010
Methionine	−1.233	0.005	Lysine	0.748	0.026	Aminoadipate	−4.066	0.043
Aspartate	1.722	0.034	Tyrosine	0.695	0.013	Proline	−0.954	0.023
Asparagine	0.836	0.001						
Tyrosine	1.438	0.000						
Glutamate	−0.360	0.001						

**Table 5:** BCAA, AAA, glutamate and glutamine comparisons between the healthy controls (G0), single-treated (G1) and multi-treated (G2) type 2 diabetes mellitus groups; data are median (interquartile range).

Amino Acid (nM)	Group									Kruskal Wallis			
	G0			G1			G2			G0 vs. G1 vs. G2	G0 vs. G1	G0 vs. G2	G1 vs. G2
	Median	Lower QR	Upper QR	Median	Lower QR	Upper QR	Median	Lower QR	Upper QR	(*p* Values)	(*p* Values)	(*p* Values)	(*p* Values)
**Isoleucine**	190.46	131.59	246.49	132.43	90.68	195.89	156.1	99	156.1	**0.018**	**0.003**	**0.003**	0.222
**Valine**	223.12	168.12	279.6	138.86	26.63	222.54	147.17	50.77	147.17	**0.000**	**0.000**	**0.000**	0.773
**Leucine**	212.14	152.71	255.4	121.47	62.43	181.85	147.64	72.43	147.64	**0.000**	**0.000**	**0.000**	0.318
**Phenyl-alanine**	138.36	77.98	193.73	77.53	54.68	170.11	163.6	59.78	163.6	**0.042**	0.058	0.058	**0.041**
**Tyrosine**	50.91	40.25	63.24	37.4	20.11	59.52	23.96	16.15	23.96	**0.000**	**0.001**	**0.001**	0.087
**Glutamate**	154.43	120.42	199	258.76	89.2	384.68	73.68	25.23	73.68	**0.002**	**0.009**	**0.009**	**0.011**
**Glutamine**	230.64	186.45	337.24	162.09	100.15	288.63	132.39	102.41	132.39	**0.000**	**0.000**	**0.000**	0.207

## Data Availability

All data underlying this article are available in the article and in its online supplementary material. We will willingly share our knowledge, protocol, and expertise when asked.

## Supplementary Materials

The following are available online at https://www.mdpi.com/article/10.3390/jcm10225424/s1, Supplementary Table S1—Amino Acid Correlations.
