# Supplementary material for: Low Branched Chain Amino Acids and Tyrosine in Thai Patients with Type 2 Diabetes Mellitus Treated with Metformin and Metformin-Sulfonylurea Combination Therapies
Source: J Clin Med. 2021 Nov 20;10(22):5424. doi: 10.3390/jcm10225424 (PMC8621185; doi:10.3390/jcm10225424)
Supplement: Supplementary file 1 [file jcm-10-05424-s001.zip › Supplementary Data S1 - Power Calculation.pdf]

Sample size was calculated using the formula below:

$$N = \frac{2 \times (Z_{1-\alpha/2} + Z_{1-\beta})^2 \times \sigma^2}{\delta^2}$$

N= number of sample (sample size), α and β error
σ = standard deviation of difference between two means
δ = difference of two means

From literature review i.e. Newgard CB, An J, Bain JR, Muehlbauer MJ, Stevens RD, Lien LF, Haqq AM, Shah SH, Arlotto M, Slentz CA, Rochon J, Gallup D, Ilkayeva O, Wenner BR, Yancy WS Jr, Eisenson H, Musante G, Surwit RS, Millington DS, Butler MD, Svetkey LP. A branched-chain amino acid-related metabolic signature that differentiates obese and lean humans and contributes to insulin resistance. Cell Metab. 2009 Apr;9(4):311-26.

$\sigma = 50$ and $\delta = 21$;
$Z_{1-\alpha/2}$ at 95% confidence levels = 1.96 and $Z_{1-\beta}$ at power of 80 % = 0.84;

$$N = \frac{2 \times (1.96 + 0.84)^2 \times 50^2}{21^2}$$

N = 89 (minimum number needed to recruit)
