# Supplementary material for: Low Branched Chain Amino Acids and Tyrosine in Thai Patients with Type 2 Diabetes Mellitus Treated with Metformin and Metformin-Sulfonylurea Combination Therapies
Source: J Clin Med. 2021 Nov 20;10(22):5424. doi: 10.3390/jcm10225424 (PMC8621185; doi:10.3390/jcm10225424)
Supplement: Supplementary file 1 [file jcm-10-05424-s001.zip › Supplementary Figure S1 - Study Flow Chart.pdf]

Study approved by the Bangkok Hospital for Tropical Diseases in Thailand certified by the Ethics Committee of the Faculty of Tropical Medicine, Mahidol University, Thailand

Study participants were recruited according to inclusion and exclusion criteria from the Hospital for Tropical Diseases, Faculty of Tropical Medicine, Mahidol University, Thailand. Written informed consent was obtained from all study participants. A total of 207 study participants were recruited comprising of individuals with T2DM (n=65 single-drug treated; n=38 multi-drug treated) and healthy controls (n=104).

Data collection from all study participants. Collection of fasting blood for laboratory analyses; separate sample of blood collected in EDTA tubes to obtain plasma that was subsequently stored at 80°C amino acids analyses by LC-MS.

Amino acids were extracted from individual plasma samples for analysis using a Phe-nomenexEZ:faast™ amino acid analysis kit; LC-MS used for simultaneous quantification of amino acids in plasma samples.

Data analyses comparing amino acid profiles across three groups: healthy controls, single-treated and multi-treated individuals with T2DM.
