# Supplementary material for: Low Branched Chain Amino Acids and Tyrosine in Thai Patients with Type 2 Diabetes Mellitus Treated with Metformin and Metformin-Sulfonylurea Combination Therapies
Source: J Clin Med. 2021 Nov 20;10(22):5424. doi: 10.3390/jcm10225424 (PMC8621185; doi:10.3390/jcm10225424)
Supplement: Supplementary file 1 [file jcm-10-05424-s001.zip › Supplementary Table S1 - Amino Acid Correlations.pdf]

Amino acid correlations

G2	G2		Isoleucine (nM)	Valine (nM)	Leucine (nM)	Phenylalanine (nM)
Spearman's rho	Isoleucine (nM)	Correlation Coefficient		.795**	.828**	.618**
		Sig. (2-tailed)		.000	.000	.000
	Valine (nM)	Correlation Coefficient	.795**		.902**	
		Sig. (2-tailed)	.000		.000	
	Leucine (nM)	Correlation Coefficient	.828**	.902**		
		Sig. (2-tailed)	.000	.000		
	Phenylalanine (nM)	Correlation Coefficient	.618**			
		Sig. (2-tailed)	.000			
	Tyrosine (nM)	Correlation Coefficient				-.425**
		Sig. (2-tailed)				.008
	Aminoadipic (nM)	Correlation Coefficient	.420**	.361*		.352*
		Sig. (2-tailed)	.009	.026		.030
	Arginine (nM)	Correlation Coefficient			.406*	
		Sig. (2-tailed)			.011	
	Glycine (nM)	Correlation Coefficient	.659**	.570**	.480**	.510**
		Sig. (2-tailed)	.000	.000	.002	.001
	Threonine (nM)	Correlation Coefficient	.474**	.326*		.488**
		Sig. (2-tailed)	.003	.045		.002
	Methionine (nM)	Correlation Coefficient	.386*	.434**	.479**	
		Sig. (2-tailed)	.017	.007	.002	
	Aspatic (nM)	Correlation Coefficient				
		Sig. (2-tailed)				
	Sacosine (nM)	Correlation Coefficient	-.517**	-.640**	-.521**	
		Sig. (2-tailed)	.001	.000	.001	
	Ornithine (nM)	Correlation Coefficient				
		Sig. (2-tailed)				
	Proline (nM)	Correlation Coefficient	.620**	.875**	.777**	
		Sig. (2-tailed)	.000	.000	.000	
	Lysine (nM)	Correlation Coefficient	-.344*			-.641**
		Sig. (2-tailed)	.034			.000

	Glutamic (nM)	Correlation Coefficient				-.506**
		Sig. (2-tailed)				.001
	Glutamine (nM)	Correlation Coefficient				
		Sig. (2-tailed)				
	Serine (nM)	Correlation Coefficient	.422**			.515**
		Sig. (2-tailed)	.008			.001
	Asparagine (nM)	Correlation Coefficient				
		Sig. (2-tailed)				
	4Hydroxyproline (nM)	Correlation Coefficient				
		Sig. (2-				

G1	G1		Isoleucine (nM)	Valine (nM)	Leucine (nM)	Phenylalanine (nM)
Spearman's rho	Isoleucine (nM)	Correlation Coefficient		.728**	.757**	.400**
		Sig. (2-tailed)		.000	.000	.001
	Valine (nM)	Correlation Coefficient	.728**		.839**	
		Sig. (2-tailed)	.000		.000	
	Leucine (nM)	Correlation Coefficient	.757**	.839**		
		Sig. (2-tailed)	.000	.000		
	Phenylalanine (nM)	Correlation Coefficient	.400**			
		Sig. (2-tailed)	.001			
	Tyrosine (nM)	Correlation Coefficient		.350**	.438**	-.370**
		Sig. (2-tailed)		.004	.000	.002
	Aminoadipic (nM)	Correlation Coefficient	.301*			.302*
		Sig. (2-tailed)	.015			.015
	Arginine (nM)	Correlation Coefficient			.300*	
		Sig. (2-tailed)			.015	
	Threonine (nM)	Correlation Coefficient	.265*			.282*
		Sig. (2-tailed)	.033			.023
	Methionine (nM)	Correlation Coefficient	.253*	.282*	.442**	
		Sig. (2-tailed)	.042	.023	.000	
	Sacosine (nM)	Correlation Coefficient	-.403**	-.337**		
		Sig. (2-tailed)	.001	.006		

	Ornithine (nM)	Correlation Coefficient				
		Sig. (2-tailed)				
	Proline (nM)	Correlation Coefficient	.670**	.667**	.555**	
		Sig. (2-tailed)	.000	.000	.000	
	Lysine (nM)	Correlation Coefficient			.327**	-.419**
		Sig. (2-tailed)			.008	.001
	Glutamic (nM)	Correlation Coefficient			.317**	-.310*
		Sig. (2-tailed)			.010	.012
	Glutamine (nM)	Correlation Coefficient			.266*	-.371**
		Sig. (2-tailed)			.033	.002
	Serine (nM)	Correlation Coefficient				.307*
		Sig. (2-tailed)				.013
	Asparagine (nM)	Correlation Coefficient				
		Sig. (2-tailed)				
	4Hydroxyproline (nM)	Correlation Coefficient				.347**
		Sig. (2-tailed)				.005
	Glycine (nM)	Correlation Coefficient	.411**	.380**		
		Sig. (2-tailed)	.001	.002		
	Aspatic (nM)	Correlation Coefficient	.347**	.307*	.359**	
		Sig. (2-tailed)	.005	.014	.004	

G0	G0		Isoleucine (nM)	Valine (nM)	Leucine (nM)	Phenylalanine (nM)
Spearman's rho	Isoleucine (nM)	Correlation Coefficient		.795**	.828**	.618**
		Sig. (2-tailed)		.000	.000	.000
	Valine (nM)	Correlation Coefficient	.795**		.902**	
		Sig. (2-tailed)	.000		.000	
	Leucine (nM)	Correlation Coefficient	.828**	.902**		
		Sig. (2-tailed)	.000	.000		
	Phenylalanine (nM)	Correlation Coefficient	.618**			
		Sig. (2-tailed)	.000			
	Tyrosine (nM)	Correlation Coefficient				-.425**
		Sig. (2-tailed)				.008

	Aminoadipic (nM)	Correlation Coefficient	.420**	.361*		.352*
		Sig. (2-tailed)	.009	.026		.030
	Arginine (nM)	Correlation Coefficient			.406*	
		Sig. (2-tailed)			.011	
	Glycine (nM)	Correlation Coefficient	.659**	.570**	.480**	.510**
		Sig. (2-tailed)	.000	.000	.002	.001
	Threonine (nM)	Correlation Coefficient	.474**	.326*		.488**
		Sig. (2-tailed)	.003	.045		.002
	Methionine (nM)	Correlation Coefficient	.386*	.434**	.479**	.091
		Sig. (2-tailed)	.017	.007	.002	.588
	Aspatic (nM)	Correlation Coefficient				
		Sig. (2-tailed)				
	Sacosine (nM)	Correlation Coefficient	-.517**	-.640**	-.521**	
		Sig. (2-tailed)	.001	.000	.001	
	Ornithine (nM)	Correlation Coefficient				
		Sig. (2-tailed)				
	Proline (nM)	Correlation Coefficient	.620**	.875**	.777**	
		Sig. (2-tailed)	.000	.000	.000	
	Lysine (nM)	Correlation Coefficient	-.344*			-.641**
		Sig. (2-tailed)	.034			.000
	Glutamic (nM)	Correlation Coefficient				-.506**
		Sig. (2-tailed)				.001
	Glutamine (nM)	Correlation Coefficient				
		Sig. (2-tailed)				
	Serine (nM)	Correlation Coefficient	.422**			.515**
		Sig. (2-tailed)	.008			.001
	Asparagine (nM)	Correlation Coefficient	.474**	.379*		.458**
		Sig. (2-tailed)	.003	.019		.004
	4Hydroxyproline (nM)	Correlation Coefficient	.321*			.462**
		Sig. (2-tailed)	.049			.004

Tyrosine (nM)	Aminoadipic (nM)	Arginine (nM)	Glycine (nM)	Threonine (nM)	Methionine (nM)	Aspatic (nM)	Sacosine (nM)	Ornithine (nM)
	.420**		.659**	.474**	.386*		-.517**	
	.009		.000	.003	.017		.001	
	.361*		.570**	.326*	.434**		-.640**	
	.026		.000	.045	.007		.000	
		.406*	.480**		.479**		-.521**	
		.011	.002		.002		.001	
-.425**	.352*		.510**	.488**				
.008	.030		.001	.002				
		.385*			.441**			.492**
		.017			.006			.002
			.528**	.593**			-.343*	
			.001	.000			.035	
.385*				-.356*				
.017				.028				
	.528**			.831**	.467**		-.399*	
	.001			.000	.003		.013	
	.593**	-.356*	.831**		.416**			
	.000	.028	.000		.009			
.441**			.467**					.334*
.006			.003					.041
	-.343*	.006	-.399*					
	.035	.972	.013					
.492**					.334*			
.002					.041			
			.568**		.539**		-.634**	.378*
			.000		.000		.000	.019
.442**		.387*						.424**
.005		.016						.008

.396*		.361*		-.347*		.420**		.421**
.014		.026		.033		.009		.008
.386*			.404*	.504**	.448**			.480**
.017			.012	.001	.005			.002
	.609**	-.439**	.813**	.952**				
	.000	.006	.000	.000				

Tyrosine (nM)	Aminoadipic (nM)	Arginine (nM)	Threonine (nM)	Methionine (nM)	Sacosine (nM)	Ornithine (nM)	Proline (nM)	Lysine (nM)
	.301*		.265*	.253*	-.403**		.670**	
	.015		.033	.042	.001		.000	
.350**				.282*	-.337**		.667**	
.004				.023	.006		.000	
.438**		.300*		.442**			.555**	.327**
.000		.015		.000			.000	.008
-.370**	.302*		.282*					-.419**
.002	.015		.023					.001
		.441**		.481**	.344**	.644**		.613**
		.000		.000	.005	.000		.000
			.286*		-.264*			
			.021		.034			
.441**				.479**				.379**
.000				.000				.002
	.286*							-.471**
	.021							.000
.481**		.479**				.315*		.332**
.000		.000				.011		.007
.344**	-.264*						-.520**	.248*
.005	.034						.000	.046

.644**				.315*				.555**
.000				.011				.000
					-.520**			
					.000			
.613**		.379**	-.471**	.332**	.248*	.555**		
.000		.002	.000	.007	.046	.000		
.440**	-.256*	.489**	-.336**	.303*	.282*			.538**
.000	.040	.000	.006	.014	.023			.000
.629**		.271*		.336**	.373**	.485**		.506**
.000		.029		.006	.002	.000		.000
	.248*	-.263*	.920**					-.575**
	.046	.034	.000					.000
	.284*		.875**					-.393**
	.022		.000					.001
-.287*	.350**		.810**					-.615**
.020	.004		.000					.000
			.657**		-.330**		.414**	-.299*
			.000		.008		.001	.016
	.295*						.356**	
	.018						.004	

Tyrosine (nM)	Aminoadipic (nM)	Arginine (nM)	Glycine (nM)	Threonine (nM)	Methionine (nM)	Aspatic (nM)	Sacosine (nM)	Ornithine (nM)
	.420**		.659**	.474**	.386*		-.517**	
	.009		.000	.003	.017		.001	
	.361*		.570**	.326*	.434**		-.640**	
	.026		.000	.045	.007		.000	
		.406*	.480**		.479**		-.521**	
		.011	.002		.002		.001	
-.425**	.352*		.510**	.488**				
.008	.030		.001	.002				
		.385*			.441**			.492**
		.017			.006			.002

			.528**	.593**			-.343*	
			.001	.000			.035	
.385*				-.356*				
.017				.028				
	.528**			.831**	.467**		-.399*	.273
	.001			.000	.003		.013	.097
	.593**	-.356*	.831**		.416**			
	.000	.028	.000		.009			
.441**			.467**	.416**	1.000			.334*
.006			.003	.009				.041
	-.343*		-.399*					
	.035		.013					
.492**					.334*			
.002					.041			
			.568**		.539**		-.634**	.378*
			.000		.000		.000	.019
.442**		.387*						.424**
.005		.016						.008
.396*		.361*		-.347*		.420**		.421**
.014		.026		.033		.009		.008
.386*			.404*	.504**	.448**			.480**
.017	#NAME?		.012	.001	.005			.002
	.609**	-.439**	.813**	.952**				
	.000	.006	.000	.000				
	.552**	-.372*	.765**	.915**	.399*			
	.000	.021	.000	.000	.013			
	.457**	-.384*	.723**	.857**				
	.004	.017	.000	.000				

Proline (nM)	Lysine (nM)	Glutamic (nM)	Glutamine (nM)	Serine (nM)	Asparagine (nM)	4Hydroxyproline (nM)
.620**	-.344*			.422**	.474**	.321*
.000	.034			.008	.003	.049
.875**					.379*	
.000					.019	
.777**						
.000						
	-.641**	-.506**		.515**	.458**	.462**
	.000	.001		.001	.004	.004
.216	.442**	.396*	.386*			
.193	.005	.014	.017			
				.609**	.552**	.457**
				.000	.000	.004
	.387*	.361*		-.439**	-.372*	-.384*
	.016	.026		.006	.021	.017
.568**			.404*	.813**	.765**	.723**
.000			.012	.000	.000	.000
		-.347*	.504**	.952**	.915**	.857**
		.033	.001	.000	.000	.000
.539**			.448**		.399*	
.000			.005		.013	
		.420**				
		.009				
-.634**						
.000						
.378*	.424**	.421**	.480**			
.019	.008	.008	.002			
		.735**	.370*	-.380*	-.327*	-.359*
		.000	.022	.019	.045	.027

	.735**		.394*	-.351*	-.367*	
	.000		.014	.031	.023	
	.370*	.394*		.426**	.442**	.454**
	.022	.014		.008	.005	.004
	-.380*	-.351*	.426**		.883**	.856**
	.019	.031	.008		.000	.000

Glutamic (nM)	Glutamine (nM)	Serine (nM)	Asparagine (nM)	4Hydroxyproline (nM)	Glycine (nM)	Aspatic (nM)
					.411**	.347**
					.001	.005
					.380**	.307*
					.002	.014
.317**	.266*					.359**
.010	.033					.004
-.310*	-.371**	.307*		.347**		
.012	.002	.013		.005		
.440**	.629**			-.287*		
.000	.000			.020		
-.256*		.248*	.284*	.350**		.295*
.040		.046	.022	.004		.018
.489**	.271*	-.263*				
.000	.029	.034				
-.336**		.920**	.875**	.810**	.657**	
.006		.000	.000	.000	.000	
.303*	.336**					
.014	.006					
.282*	.373**				-.330**	
.023	.002				.008	

	.485**					
	.000					
					.414**	.356**
					.001	.004
.538**	.506**	-.575**	-.393**	-.615**	-.299*	
.000	.000	.000	.001	.000	.016	
	.385**	-.390**	-.262*	-.446**		.337**
	.002	.001	.035	.000		.006
.385**			.325**			
.002			.008			
-.390**			.871**	.816**	.650**	
.001			.000	.000	.000	
-.262*	.325**	.871**	1.000	.717**	.551**	
.035	.008	.000		.000	.000	
-.446**		.816**	.717**		.597**	
.000		.000	.000		.000	
		.650**	.551**	.597**		
		.000	.000	.000		
.337**						
.006						

Proline (nM)	Lysine (nM)	Glutamic (nM)	Glutamine (nM)	Serine (nM)	Asparagine (nM)	4Hydroxyproline (nM)
.620**	-.344*			.422**	.474**	.321*
.000	.034			.008	.003	.049
.875**					.379*	
.000					.019	
.777**						
.000						
	-.641**	-.506**		.515**	.458**	.462**
	.000	.001		.001	.004	.004
	.442**	.396*	.386*			
	.005	.014	.017			

				.609**	.552**	.457**
				.000	.000	.004
	.387*	.361*		-.439**	-.372*	-.384*
	.016	.026		.006	.021	.017
.568**			.404*	.813**	.765**	.723**
.000			.012	.000	.000	.000
		-.347*	.504**	.952**	.915**	.857**
		.033	.001	.000	.000	.000
.539**			.448**			
.000			.005			
		.420**				
		.009				
-.634**						
.000						
.378*	.424**	.421**	.480**			
.019	.008	.008	.002			
		.735**	.370*	-.380*	-.327*	-.359*
		.000	.022	.019	.045	.027
	.735**	1.000	.394*	-.351*	-.367*	
	.000		.014	.031	.023	
	.370*	.394*		.426**	.442**	.454**
	.022	.014		.008	.005	.004
	-.380*	-.351*	.426**	1.000	.883**	.856**
	.019	.031	.008		.000	.000
	-.327*	-.367*	.442**	.883**		.827**
	.045	.023	.005	.000		.000
	-.359*		.454**	.856**	.827**	
	.027		.004	.000	.000	
